# A Dinuclear Copper(II) Complex Electrochemically Obtained via the Endogenous Hydroxylation of a Carbamate Schiff Base Ligand: Synthesis, Structure and Catalase Activity

**DOI:** 10.3390/ijms25042154

**Published:** 2024-02-10

**Authors:** Sandra Fernández-Fariña, Isabel Velo-Heleno, Laura Rodríguez-Silva, Marcelino Maneiro, Ana M. González-Noya, Rosa Pedrido

**Affiliations:** 1 Departamento de Química Inorgánica, Facultade de Ciencias, Campus Terra, Universidade de Santiago de Compostela, 27002 Lugo, Spain; sandra.fernandez.farina@usc.es (S.F.-F.) laura.rodriguez@usc.es (L.R.-S.); marcelino.maneiro@usc.es (M.M.); 2 Departamento de Química Inorgánica, Facultade de Química, Campus Vida, Universidade de Santiago de Compostela, 15782 Santiago de Compostela, Spain; mariaisabel.velo.heleno@usc.es (I.V.-H.); ana.gonzalez.noya@usc.es (A.M.G.-N.)

**Keywords:** Schiff bases, copper, catalase activity, hydroxylation

## Abstract

In the present work, we report a neutral dinuclear copper(II) complex, [Cu_2_(L^1^)(OH)], derived from a new [N,O] donor Schiff base ligand L^1^ that was formed after the endogenous hydroxylation of an initial carbamate Schiff base H_2_L coordinated with copper ions in an electrochemical cell. The copper(II) complex has been fully characterized using different techniques, including X-ray diffraction. Direct current (DC) magnetic susceptibility measurements were also performed at variable temperatures, showing evidence of antiferromagnetic behavior. Its catalase-like activity was also tested, demonstrating that this activity is affected by temperature.

## 1. Introduction

Living aerobic organisms, which depend on oxygen to obtain metabolic energy in the form of ATP, also produce Reactive Oxygen Species (ROS), whose reactivity and cytotoxic potential must be strictly controlled, as they can become harmful if their concentration inside cells exceeds a limiting threshold, leading to oxidative stress (OS) [1,2,3]. The most reactive ROS are superoxide anions (O_2_^−^), hydroxyl radicals (OH^•^), singlet oxygen and hydrogen peroxide (H_2_O_2_). These radical species are involved in cell damage, to the extent that oxidative stresses can lead to carcinogenesis, inflammatory diseases, cellular senescence and neurodegenerative diseases, among other pathological processes [4,5,6].

ROS control in organisms is carried out by a molecular army known as antioxidants. Antioxidant defenses include stoichiometric scavengers such as ascorbic acid (vitamin C), glutathione and metalloenzymes like superoxide dismutase (SOD) [7,8,9,10,11,12], catalase (CAT) [13,14], glutathione peroxidase (GPx) and glutathione reductase (GR) [15,16].

Catalase (hydrogen peroxide:hydrogen peroxide oxidoreductase) is one of the most abundant enzymes in plants and mammals, being widely distributed in the human body, although its activity varies depending on the tissue. These enzymes are highly effective antioxidant species that convert hydrogen peroxide into water and oxygen, preventing its accumulation in the cells and protecting the organism from these reactive species.

To date, some small-size inorganic complexes mimicking CAT have been developed in laboratories to reduce the oxidative stress in pathological conditions [17,18,19]. These mimetic complexes are usually mononuclear iron, manganese derivatives or dinuclear manganese species [19]. In this sense, very few copper complexes [18,20,21,22,23,24] have been reported to display CAT activity. Although the reaction of hydrogen peroxide dismutation via catalase mimics requires two electrons, in some cases, monomeric complexes can catalyze this reaction. For example, ligands such as porphyrin can be oxidized and participate in the redox process. In the case of non-heme metal complexes, the bi-electronic process can be facilitated by increasing their nuclearity. Two metal ions with the ability to each undergo mono-electronic redox processes may properly catalyze H_2_O_2_ dismutation. However, dinuclear copper complexes with CAT activity are very rare [25,26,27,28]. In 2021, the first dinuclear copper(II) complex with catalase activity was published [29]. Also, Cu(I) and Cu(II) complexes with enzymatic activity capable of promoting exogenous monohydroxylation or intramolecular double hydroxylation were found in [30].

In recent decades, our research group has investigated how to model different natural enzymes with synthetic complexes derived from salen-type Schiff base ligands. In parallel, our group is specialized in the design of suitable ligands to generate supramolecular helical or meso-helical complexes. In the context of both areas of research, we designed a Schiff base ligand with two carbamate [NO] binding domains separated by a phenyl spacer as a potential precursor of dinuclear complexes. It should be noted that our research group previously published a copper(II) helicate derived from a related ligand that incorporates pyridine into the spacer instead of phenyl [31].

In this paper, we report the synthesis, characterization and catalase activity of a new dinuclear copper(II) complex obtained after an endogenous hydroxylation process using a carbamate Schiff base ligand in an electrochemical cell. The magnetic behavior and the CAT activity of the complex have been tested in the search of new catalase mimics for medical or industrial applications.

## 2. Results and Discussion

### 2.1. Synthesis and Characterization of the Schiff Base Ligand H_2_L

**Figure 1:**
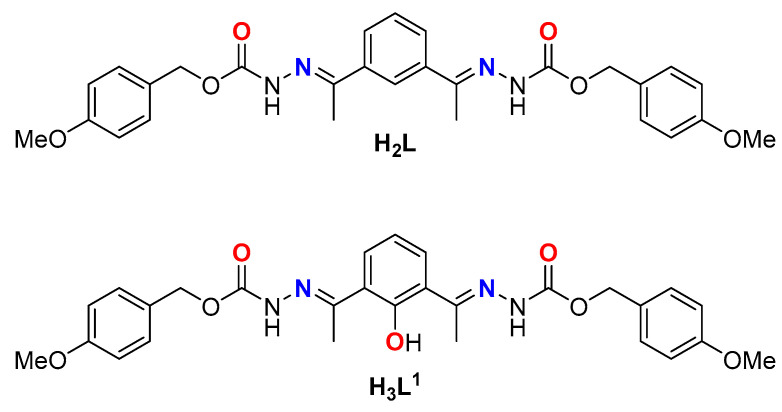
Schiff base ligands: H_2_L synthetized ligand (**top**); hydroxylated derivative ligand H_3_L^1^ (**bottom**).

In the present work, we have designed a potentially dianionic Schiff base ligand H_2_L functionalized with carbamate groups separated by a rigid benzene spacer, providing a tetradentate [N_2_O_2_] system (Figure 1, top). It was demonstrated that different ligands with benzene-derived spacers are potentially precursors of dinuclear metallosupramolecular structures using several metal ions [32]. It should be noted that the ligand H_2_L undergoes a hydroxylation process, giving rise to a new derived pentadentate [N_2_O_3_] system, H_3_L^1^ (Figure 1, bottom).

The Schiff base ligand H_2_L was synthetized according to the reaction between 1,3-diacetylbenzene and 4-methoxybenzylcarbazate (1:2 ratio), using a catalytic amount of *p-*toluensulfonic acid and absolute ethanol as the solvent. Once isolated, H_2_L was fully characterized using different techniques: melting point determination, elemental analysis, infrared spectroscopy, mass spectrometry and ^1^H and ^13^C NMR spectroscopy (Figures S1–S5, Supplementary Materials).

### 2.2. Synthesis and Characterization of the Copper Complex

A neutral copper complex was isolated from the Schiff base ligand H_2_L using an electrochemical synthesis methodology (see details in the Section 3). The resulting green solid was characterized in both its solid state and solution using melting point determination, elemental analysis, infrared spectroscopy, room-temperature magnetic susceptibility, X-ray diffraction studies and mass spectrometry, thus demonstrating its purity and stability.

**Figure 2:**
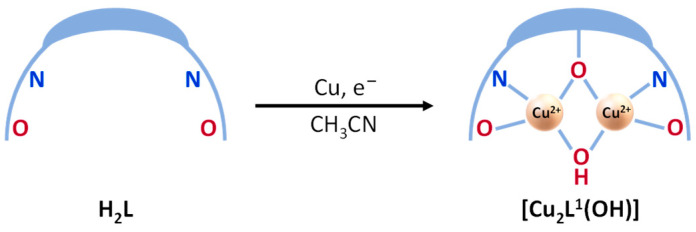
Hydroxylation process of the Schiff base ligand H_2_L during the electrochemical synthesis of the copper(II) complex.

The structure of the copper complex determined using X-ray diffraction showed that the initial ligand H_2_L underwent a hydroxylation process at the central carbon atom of the benzene spacer, giving rise to a new potentially trianionic Schiff base ligand, H_3_L^1^ (Figure 2). Furthermore, the characterization techniques allowed us to suggest a dinuclear [Cu_2_(L^1^)(OH)] stoichiometry, with the ligand coordinated with both metal ions in a trianionic form [L^1^]^3−^. The coordination sphere of the copper(II) metal ions was completed with an exogenous hydroxyl group.

The infrared spectrum of the complex (Figures S6 and S7) shows the absence of the characteristic bands of the NH groups, proving the deprotonation of the ligand when coordinating with metal ions. Also, the appearance of a band at around 3400 cm^−1^ confirms the presence of the OH group from the µ_2_-OH bridge in the dinuclear copper complex. Likewise, the ν(C=N) band has undergone a slight shift, indicating that the imine nitrogen is involved in coordinating the metal ions. The bands involving the amide group, amide I and amide II, show a slight shift in the complex. This is compatible with the deprotonation of the NH groups by the amide–imidol tautomerism. The formation of the dinuclear complex derived from the Schiff base ligand H_3_L^1^ was also confirmed using the ESI^+^ mass spectrometry technique (Figure S8), as the peak corresponding to the dinuclear fragment [Cu_2_(L^1^)(OH) + H]^+^ is observed in the mass spectra.

#### X-ray Structure

**Figure 3:**
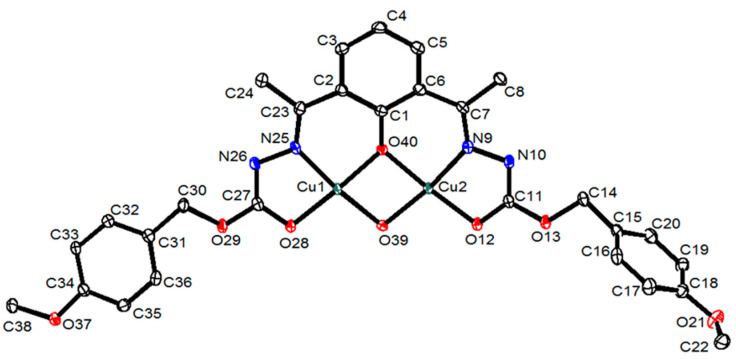
ORTEP representation of the dinuclear neutral copper(II) complex [Cu_2_(L^1^)(OH)]·2CH_3_CN. Solvent molecules and hydrogen atoms were omitted for clarity.

Slow evaporation of the mother liquors from the electrochemical synthesis of the copper complex yielded green crystals suitable for single-crystal X-ray diffraction studies. The crystal structure of the formula [Cu_2_(L^1^)(OH)]·2CH_3_CN is depicted in Figure 3. Table S1 contains the main crystallographic data for the complex, while Table S2 summarizes the most relevant distances and angles.

The complex crystallizes as discrete [Cu_2_(L^1^)(OH)]·2CH_3_CN molecules. Each molecule of the newly formed ligand [L^1^]^3−^ coordinates with two Cu(II) ions via a [N_2_O_3_] donor system (Figure 3), consisting of the imine nitrogen atoms, the amide oxygen atoms and a phenolic oxygen atom formed in the intramolecular oxidation process. Each of the Cu(II) ions in the complex is coordinated with an imine nitrogen atom and an amide oxygen atom of the [L^1^]^3−^ ligand (Figure 3). In addition, two bridges between both metal centers are formed by phenolic and hydroxyl oxygen atoms, giving rise to the distorted square planar geometry of the metal ions. The O-M-N bond angles show the distortion of the square planar geometry (Table S2). As mentioned in the introduction, our research group previously published a copper(II) helicate derived from a related ligand that incorporated pyridine into the spacer in place of benzene, obtained using the same electrochemical methodology [31]. The isolation of [Cu_2_(L^1^)(OH)]· instead of the expected helicate [Cu_2_L_2_] demonstrated that the use of a phenyl spacer rather than pyridine is the key factor that allows the hydroxylation process to occur, avoiding the formation of a dinuclear helicate.

The values of the bond distances (Table S2) between the metal centers and the imine nitrogen atoms, Cu(1)-N(25) and Cu(2)-N(9) [1.9105(19) and 1. 9145(19) Å, respectively], as well as the distances between the copper atoms and the amide oxygen atoms, Cu(1)-O(28) and Cu(2)-O(12) [1.9185(15) and 1.9524(15) Å], are in the order of those found in other complexes with Schiff base hydrazone ligands [30,33,34]. The bond distances of the copper atoms to the μ_2_-oxo oxygen atoms of the spacer [Cu(1)-O(40) and Cu(2)-O(40) 1.9234(15) and 1.9234(15) and 1. 9498(15) Å, respectively] and those for the μ_2_-OH bridge oxygen atoms [Cu(1)-O(39) and Cu(2)-O(39) 1.9104(17) and 1.9371(17) Å, respectively] are similar to those found in the literature for similar coordination environments [35,36,37,38].

**Figure 4:**
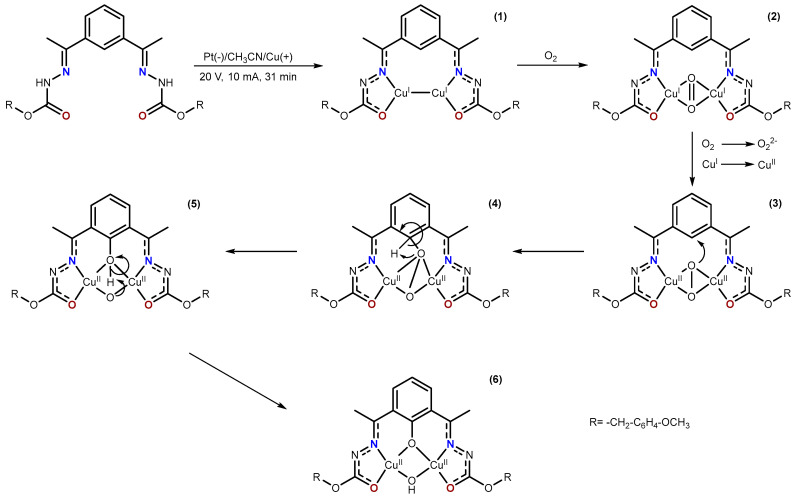
Proposed mechanism of formation of [Cu_2_(L^1^)(OH)].

The formation of this complex according to the precedents found in the literature can be explained by a six-step reaction mechanism (Figure 4) [39]. First, the electrochemical synthesis of the Cu(I) complex takes place (**1**), which, upon interaction with the O_2_ present in the reaction medium, leads to the formation of a Cu(I)-O_2_-Cu(I) adduct (**2**). Then, the oxidation reaction of the Cu(I) complex due to the molecular O_2_ takes place (E^0^= 0.463 V). In this step, the O=O double bond is broken, which favors the intramolecular attack of the oxygen atom on the aromatic carbon of the spacer (**3**). The leaving group, the aromatic hydrogen atom, is initially transferred to the oxygen attached to the aromatic ring (**4**), to be transferred back to the other oxygen atom µ_2_ coming from the O_2_ molecule, which gives rise to the hydroxyl co-ligand (**5**). The result is the formation of the complex [Cu_2_(L^1^)(OH)], which contains the new pentadentate [L^1^]^3−^ ligand (**6**).

**Figure 5:**
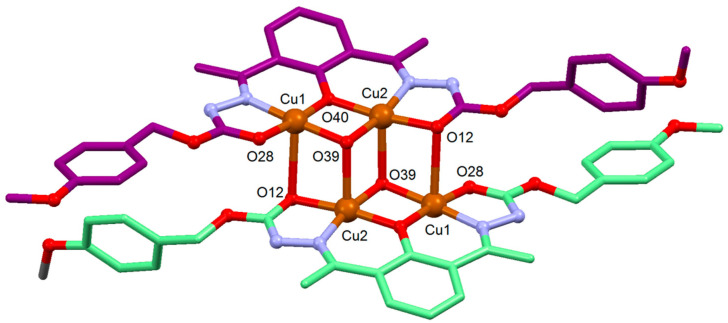
Pseudo-tetranuclear structure of the [Cu_2_(L^1^)(OH)]·2CH_3_CN complex.

Each complex molecule establishes weak contact with the atoms of a neighboring complex molecule: one of the Cu(II) centers is bonded to a μ_2_-OH oxygen atom [Cu(2)-O(39) 1.9371(17) Å], whereas the other metal center coordinates with an amide oxygen atom [Cu(1)-O(12) 2.9731(9) Å] (Figure 5). Taking this weak contact into account, the coordination geometry could be considered a square-based pyramid (τ_1_ = 0.007; τ_2_ = 0.064). The base of the pyramid would be formed by the two amide oxygen atoms, with the imine nitrogen and the phenolic and hydroxyl oxygens both acting as a bridge, while the apical positions of the Cu(1) and Cu(2) environments would be occupied by the amide oxygen atoms [O(12)], respectively, of a neighboring complex molecule. The Cu(II) ions would be located in the center of the pyramid base.

**Figure 6:**
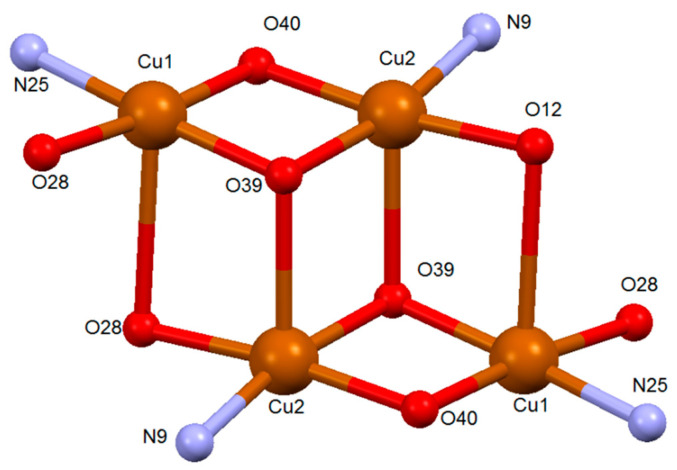
Core of the tetranuclear entity [Cu_2_(L^1^)(OH)]·2CH_3_CN.

In the crystal structure of the [Cu_2_(L^1^)(OH)]·2CH_3_CN complex (Figure 5), the ligand molecules are arranged in an antiparallel fashion, leading to the formation of a pseudo-cubic Cu(II) ion cluster (Figure 6), as previously found in the literature in several papers, where these types of hydroxo:phenoxo bridges connect two Cu(II) ions [37,40,41,42].

**Figure 7:**
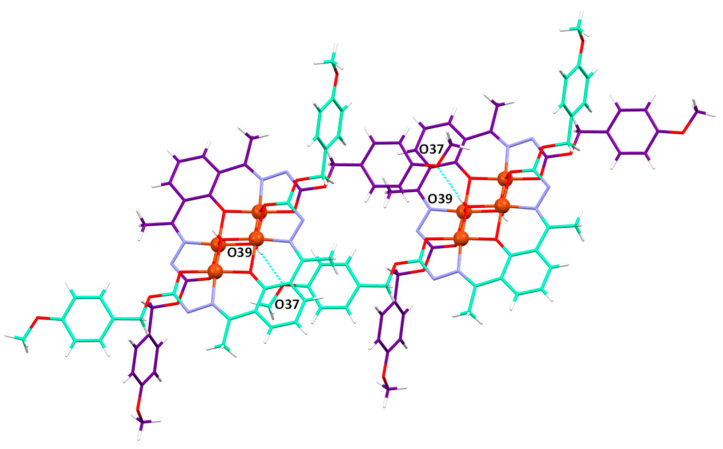
Intermolecular hydrogen bonds in [Cu_2_(L^1^)(OH)]·2CH_3_CN.

In the crystal lattice of the [Cu_2_(L^1^)(OH)]·2CH_3_CN complex, intermolecular hydrogen bonds are established between the methoxyphenyl groups on each pseudo-tetramer and the μ_2_-OH group of a neighboring tetramer. These interactions result in the 3D packing of the tetramer chains (Figure 7).

### 2.3. Magnetic Studies

The magnetic moment of the complex [Cu_2_(L^1^)(OH)] was measured at room temperature, giving rise to a value of 1.8 M.B. This value is lower than that expected for a magnetically diluted d^9^ copper(II) system. The presence of two copper(II) ions located at a short distance from each other and bridged by oxygen atoms suggests interesting magnetic behaviors that deserve to be studied.

**Figure 8:**
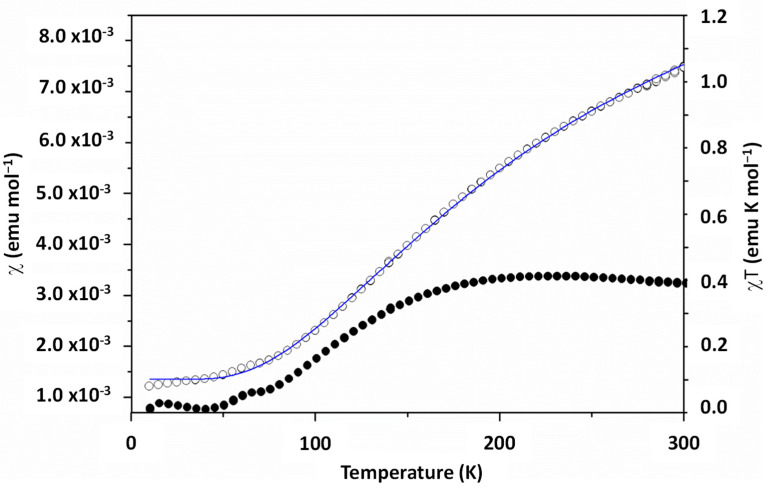
Dependence of χ (black, left axis) and χT (blue, right axis) on T/K for the [Cu_2_(L^1^)(OH)] complex.

The magnetic studies on [Cu_2_(L^1^)(OH)] were carried out at variable temperature, using a magnetic field of 1000 Oe in the temperature range of 5–300 K. The complex [Cu_2_(L^1^)(OH)] shows evidence of antiferromagnetic behavior, with strong coupling between the two Cu(II) centers (Figure 8). Based on magnetic–structural considerations [43] and the literature on complexes with structurally similar magnetic cores [44,45,46], this is the expected behavior given the value of the Cu(1)-O(40)-Cu(2) [98.01(7)^0^] and Cu(1)-O(39)-Cu(2) [98.89(2)^0^] angles, and also taking into account the correlation proposed by Hatfield and Hodgson, according to which a larger Cu-O-Cu angle leads to stronger antiferromagnetic coupling [47,48].

Up to approximately 50 K, the complex is in a singlet state. Above this temperature, the value of χ starts to increase due to the increase in the population of the excited triplet state, passing through an anchored maximum at around 230 K. Fitting the data using the Bleaney–Bowers equation (χ_A_ = Ng^2^B^2^/3kT[1 + 1/3·e^J/kT^]^−1^ + N_α_) for the Cu(II) dimers (S = ^1^/_2_) gives a best fit value for J = 240 ± 5 cm^−1^ (R^2^ = 0.99987).

### 2.4. Catalase Activity of the Copper(II) Complex

With the aim of exploring whether the copper(II) complex [Cu_2_(L^1^)(OH)] could be a good model of artificial enzymes, its catalase-like activity was explored. In this sense, we evaluated the dismutation rate of H_2_O_2_ when catalyzed by [Cu_2_(L^1^)(OH)] by volumetric determining the released O_2_. To perform this experiment, the copper(II) complex was dissolved in DMF. The experiment was carried out measuring the dioxygen evolved using H_2_O_2_ (2.5 M) at 23 °C (catalytic cycles: 113 ± 1). The catalase activity observed for the copper complex shows a hydrogen peroxide decomposition percentage of 34.2%.

Additionally, in order to study the dependence between the catalytic behavior of the complex and the concentrations of H_2_O_2_ used, different experiments were carried out varying the peroxide concentration but keeping constant the complex concentration. These probes were also carried out at two different temperatures (room temperature, 23 °C; body temperature, 36 °C).

**Figure 9:**
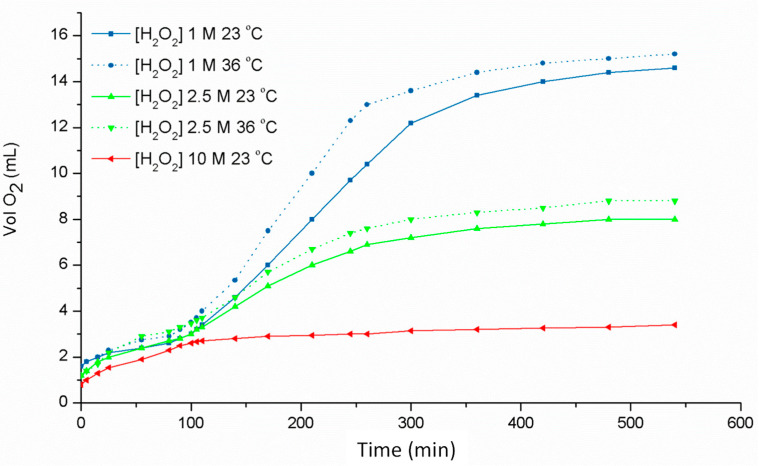
Graphical evolution of the dioxygen released by the [Cu_2_(L^1^)(OH)] complex in DMF at 23 °C and 36 °C.

Table 1 shows the data obtained on the oxygen released and the number of catalytic cycles for the catalase reaction under the different experimental conditions. All the measurements were performed in triplicate, and the limits of error derived from the different measurements are detailed in the table. The reaction is finished after 600 min since, after this time, no dioxygen evolution is observed. Figure 9 shows the evolution of the oxygen released in the first 580 min of the process using different concentrations of hydrogen peroxide (10, 2.5 and 1 M) at 23 °C and 36 °C.

This study shows how the yield of the catalytic reaction increases dramatically when decreasing the H_2_O_2_ concentration: from around 18% for 10 M H_2_O_2_ to over 100% for 1 M H_2_O_2_. The values can be easily contrasted in Table 1, which lists the number of observed experimental catalytic cycles and the yield for the complex, arranged in decreasing order of H_2_O_2_ concentration.

Moreover, studying the catalase activity of the complex at different temperatures, 23 °C and 36 °C, shows a higher catalase activity at higher temperatures. In the tests carried out, 36 °C was chosen because it is the temperature found in some biological environments (e.g., body temperature in humans) and 23 °C chosen as a reference for room temperature. Despite the relatively small difference in temperature, significantly higher activities are achieved at 36 °C.

The copper(II) complex [Cu_2_(L^1^)(OH)] shows a high catalase activity compared to other Cu(II) complexes [49,50,51,52]. The trend in the dioxygen release in the reaction catalyzed by [Cu_2_(L^1^)(OH)] shows a slower release of oxygen during the first 80 min. At that point, the oxygen production is increased during the following 200 min, until finally reaching stable levels (Figure 9). This behavior has been found in catalase studies of some complexes. However, in our case, the volumes of released gas exceed the theoretical value of the dioxygen from the added hydrogen peroxide. The reaction was repeated four times, validating the results obtained, which reached 122.9% of O_2_ at 36 °C.

## 3. Materials and Methods

All the solvents (LABKEM, Lucknow, India), *p*-toluensulfonic acid (Sigma-Aldrich, St. Louis, MO, USA), 4-methoxyphenylcarbazate (Fisher Scientific, Hampton, NH, USA), 1,3-diacetylbenzene (Sigma-Aldrich) and a copper plate (Sigma-Aldrich) were purchased from commercial sources and were used without purification. The melting points were determined using a BUCHI 560 instrument. Elemental analysis of the compounds (C, N and H) was carried out using a FISONS EA model 1108 analyzer. The infrared spectra were recorded from 4000 to 500 cm^−1^ using a Bruker FT-MIR spectrophotometer, model VERTEX 70v, in the solid state using KBr pellets. The mass spectra were obtained using Bruker micrOTOF spectrometer for the ESI^+^ technique (electrospray ionization in positive mode) and the Bruker autoflex for the MALDI technique (matrix-assisted laser desorption/ionization), both coupled with a time-of-flight (TOF) analyzer. A Varian INOVA 400 spectrometer was employed to record the ^1^H and ^13^C NMR spectra, operating at room temperature using dmso-d_6_ as deuterated solvent. The chemical shifts are reported as *δ* (in ppm).

### 3.1. Synthesis and Characterization of the Schiff Base Ligand H_2_L

H_2_L: A total of 0.2 g (1.24 mmol) of 1,3-diacetylbenzene was solved in 250 mL of absolute ethanol with a catalytic amount of *p*-toluensulfonic acid. Then, 0.49 g (2.48 mmol) of 4-methoxybenzylcarbazate was added. The reaction mixture was heated under reflux with magnetic stirring for 4 h, using a Dean–Stark manifold to remove water and promote ligand formation. The resulting solution was cooled, resulting in the formation of a white precipitate that was filtered off and air-dried. Yield: 0.45 g (71%); m.p.: 165 °C; elemental analysis: % (C_28_H_30_N_4_O_6_) C, 67.8; N, 6.0; H, 12.4; experimental C, 67.9; N, 5.9; H, 12.3; IR (cm^−1^) ν: 3275 m (N-H), 1705 s (C=O), 1610 m (C=N), 1514 s δ (NH) + (N-CO), 1248 s δ (NH) + (C−O), 1030 s (N-N); ESI(-) (*m*/*z*) 517.2 [H_2_L-H]^−^; ^1^H-NMR (400 MHz, dmso-d_6_, δ (m, nH, Hx, *J*)): 10.24 (s, 2H, H1), 8.03 (s, 2H, H2), 7.70 (d, 2H, H3, *J* = 7.8 Hz), 7.39–7.35 (t + d, 1H + 4H, H4 + H5, *J*1 = 7.8 Hz, *J*2 = 8.6 Hz), 6.90 (d, 2H, H6, *J* = 8.6 Hz), 5.11 (s, 4H, H7), 3.74 (s, 6H, H8), 2.21 (s, 6H, H9). ^13^C-NMR (400 MHz, dmso-d_6_, δ 159.5 (C=O), 154.5 (C_ar_-O), 149.5 (C=N), 138.8 (C_ar_), 130.5 (C_ar_), 128.9 (CH_ar_), 128.7 (CH_ar_), 127.0 (CH_ar_), 124.0 (CH_ar_), 114.2 (CH_ar_), 66.4 (CH_2_), 55.3 (OCH_3_), 14.2 (CH_3_).

### 3.2. Synthesis and Characterization of the Dinuclear Neutral Copper(II) Complex

The dinuclear neutral copper(II) complex was obtained employing an electrochemical methodology using acetonitrile as the solvent and applying a current intensity of 10 mA and a potential value of 20 V. The procedure is described below:

The electrochemical cell can be denoted as Pt(-)|H_2_L+ CH_3_CN|Cu(+). The Schiff base ligand H_2_L (0.05 g, 0.096 mmol) was previously dissolved in acetonitrile (80 mL), and a small amount of tetraethylammonium perchlorate was added to act as a conducting electrolyte. The electrolytic reaction was carried out under a N_2_(g) atmosphere at 10 mA and 20.0 V for 31 min. The resulting solution was concentrated, giving rise to a green solid that was filtered off, washed with acetonitrile and dried with diethyl ether. Caution: Although perchlorate salts were used in very small quantities in these reactions, they are potentially explosive and should be used with care.

[Cu_2_(L^1^)(OH)]: green solid. Yield: 0.050 g (77%); m.p.: >300 °C; Ef = 0.8 mol·F^−1^; elemental analysis: % theoretical (C_28_H_28_N_4_O_8_Cu_2_) C, 49.8; N, 8.3; H, 4.2; experimental C, 49.4; N, 4.0; H, 8.4; IR (cm^−1^) ν: 3473 m (OH), 1707 w (C=O), 1612 m (C=N), 1512 ms δ (NH) + (N-CO), 1240 s δ (NH) + (C−O), 1028 s (N-N); MS ESI(+) (*m*/*z*): 675.8 [M_2_(L^1^)(OH)+H]^+^, 1191.6 [M_2_(L^1^)_2_+H]^+^, 1354.7 [M_4_(L^1^)_2_(OH)_2_+H]^+^. Λ_M_ (μS cm^2^) = 1.8; μ (B. M.) = 1.9. Through slow evaporation of the mother liquors from the synthesis of the complex in acetonitrile, green crystals suitable for X-ray diffraction studies were obtained [Cu_2_(L^1′^)(OH)]·2CH_3_CN.

### 3.3. X-ray Crystallography

The crystallographic data for the copper(II) complex [Cu_2_(L^1^)(OH)]·2CH_3_CN were collected at 100 K using a Bruker APEX II diffractometer with a CCD area detector, using graphite monochromated MoK(α) radiation (λ = 0.71073 Å). The data were treated using the APEX2 software, version 2.0 [53]. An empirical absorption correction (SADABS) [54] was applied to the collected reflections.

The structure was solved using the SIR-97 program [55] and refined using the full-matrix least-squares technique against F2 using SHELXL-97 [55,56]. All the structures were refined using SHELXL1997 [57]. The hydrogen atoms were included in the model in geometrically calculated and refined positions. The images included in this manuscript were prepared using ORTEP3 [58] and Mercury [59]. CCDC no. 2327631 contains the supplementary crystallographic data for this compound.

### 3.4. Magnetic Susceptibility Measurements

Direct current magnetic susceptibility measurements for the copper(II) complex [Cu_2_(L^1^)(OH)] were carried out using a Quantum Design PPMS susceptometer. The magnetic susceptibility data were recorded under a magnetic field of 1000 Oe in a temperature range of 5–300 K.

### 3.5. Catalase Activity of the Copper(II) Complex

A 10 mL flask with 3 mL of a solution of the copper(II) complex [Cu_2_(L^1^)(OH)] in DMF (1 × 10^−3^ M) was sealed with a septum so that there was no loss of gas. Then, the flask was connected to a 25 mL gas measuring burette (accuracy 0.2 mL). Catalysis was initiated by the introduction of H_2_O_2_ solution (1 mL, 2.5 M) with a syringe into the test tube containing the complex. The solution was stirred at constant temperature, and the evolved dioxygen (O_2_) was volumetrically measured every 5 min.

## 4. Conclusions

We report the synthesis of a dinuclear complex [Cu_2_(L^1^)(OH)], formed after an endogenous arene hydroxylation process using the carbamate Schiff base ligand H_2_L in an electrochemical cell. The probable mechanism that explains the isolation of the complex involves the coordination of the Cu(I) ions with the H_2_L and the scavenging of oxygen from the reaction media. Direct current (DC) magnetic measurements at variable temperatures show evidence of antiferromagnetic behavior. The catalase-like activity was also tested, demonstrating that this activity is affected by temperature. We can state that the complex [Cu_2_(L^1^)(OH)] shows an important catalase-like activity, although further studies are necessary. This approach could open up new perspectives for the design of copper complexes with antioxidant properties.

## Figures and Tables

**Table 1:** H_2_O_2_ decomposition rates and number of catalytic cycles involved in the catalase activity of the [Cu_2_(L^1^)(OH)] complex in DMF (580 min).

Concentration	Temperature	Catalase Activity	Catalytic Cycles
10 M	23 °C	17.9%	60 ± 6
2.5 M	23 °C	34.2%	113 ± 1
2.5 M	36 °C	54.9%	182 ± 2
1 M	23 °C	110.4%	366 ± 1
1 M	36 °C	122.9%	408 ± 1

## Data Availability

The crystallographic data for [Cu_2_(L^1^)(OH)]·2CH_3_CN were deposited into the Cambridge Crystallographic Data Centre, CCDC 2327631. These data can be obtained free of charge via www.ccdc.cam.ac.uk/data_request/cif, (accessed on 7 January 2024) by emailing data_request@ccdc.cam.ac.uk or by contacting The Cambridge Crystallographic Data Centre at 12 Union Road, Cambridge, CB2 1EZ, the UK; Fax: +44-1223-336033.

## Supplementary Materials

The supporting information can be downloaded at https://www.mdpi.com/article/10.3390/ijms25042154/s1.
