# Supplementary material for: A Dinuclear Copper(II) Complex Electrochemically Obtained via the Endogenous Hydroxylation of a Carbamate Schiff Base Ligand: Synthesis, Structure and Catalase Activity
Source: Int J Mol Sci. 2024 Feb 10;25(4):2154. doi: 10.3390/ijms25042154 (PMC10889102; doi:10.3390/ijms25042154)
Supplement: Supplementary file 1 [file ijms-25-02154-s001.zip › ijms-2867954-supplementary.pdf]

International Journal of *Molecular Sciences*

MDPI

Supporting Information

A Dinuclear Copper(II) Complex Electrochemically Obtained via the Endogenous Hydroxylation of a Carbamate Schiff Base Ligand: Synthesis, Structure and Catalase Activity

Sandra Fernández-Fariña [1], Isabel Velo-Heleno [2], Laura Rodríguez-Silva [1], Marcelino Maneiro [1], Ana M. González-Noya [2] and Rosa Pedrido [2,*]

[1] Departamento de Química Inorgánica, Facultade de Ciencias, Campus Terra, Universidade de Santiago de Compostela, 27002 Lugo, Spain; sandra.fernandez.farina@usc.es (S.F.-F.) laura.rodriguez@usc.es (L.R.-S.); marcelino.maneiro@usc.es (M.M.)

[2] Departamento de Química Inorgánica, Facultade de Química, Campus Vida, Universidade de Santiago de Compostela, 15782 Santiago de Compostela, Spain; mariaisabel.velo.heleno@usc.es (I.V.-H.); ana.gonzalez.noya@usc.es (A.M.G.-N.)

* Correspondence: rosa.pedrido@usc.es

1. Schiff base ligand H_2L

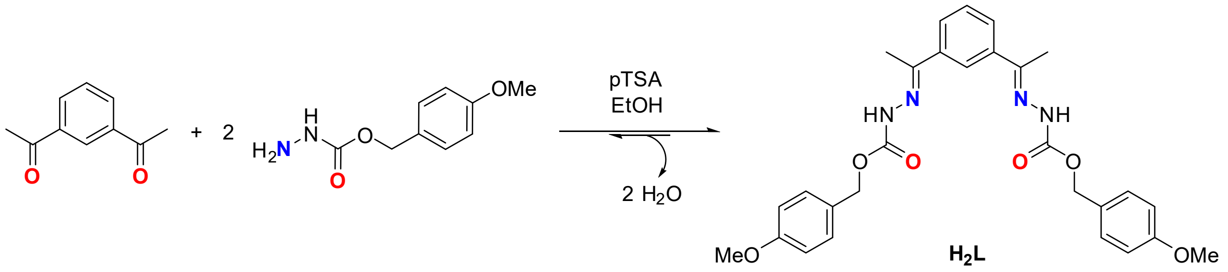

Figure S1. Synthesis of the Schiff base ligand H_2L

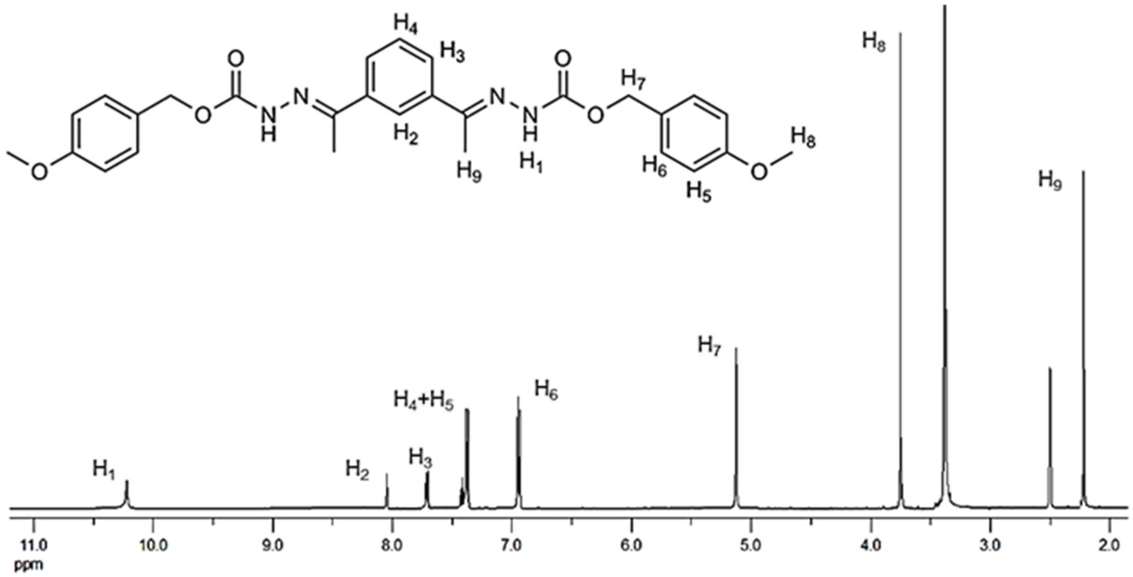

Figure S2. ^{1}H NMR spectra of H_2L (400 MHz, DMSO-d_6, r.t., δ (m, nH, Hx, J)): 10.24 (s, 2H, H_1), 8.03 (s, 2H, H_2), 7.70 (d, 2H, H_3, J= 7.8 Hz), 7.39-7.35 (t+d, 1H+4H, H_4+H_5, J1= 7.8 Hz, J2= 8.6 Hz), 6.90 (d, 2H, H_6, J= 8.6 Hz), 5.11 (s, 4H, H_7), 3.74 (s, 6H, H_8), 2.21 (s, 6H, H_9).

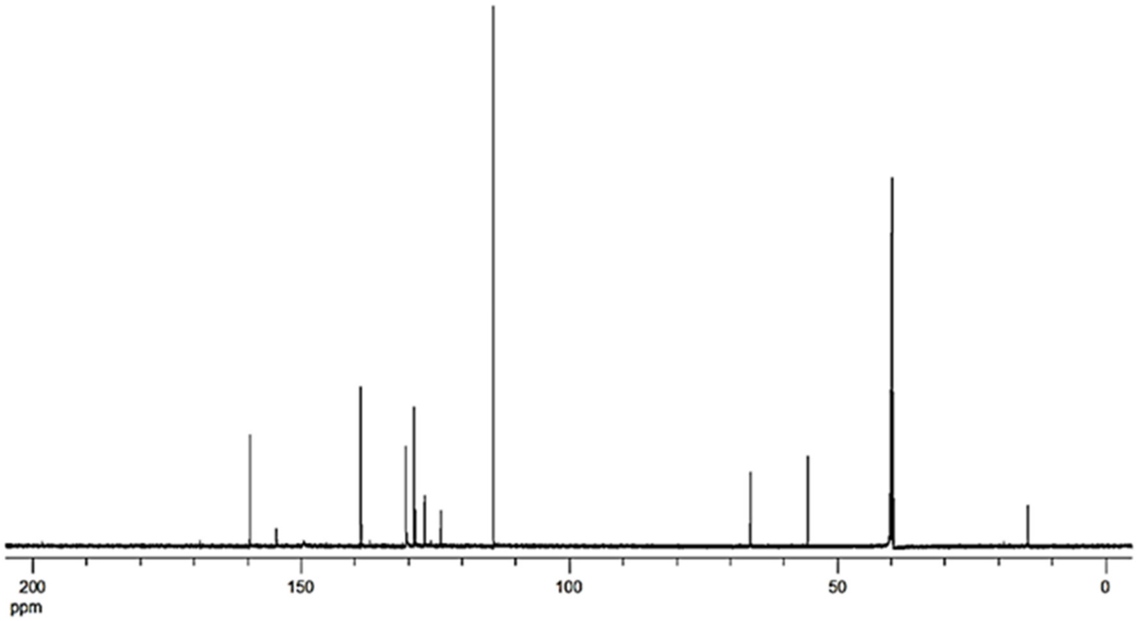

Figure S3. ^{13}C NMR spectra of H_2L (400 MHz, DMSO-d_6, r.t., δ (m, nH, Hx, J)): 159.5 (C=O), 154.5 (Car-O), 149.5 (C=N), 138.8 (Car), 130.5 (Car), 128.9 (CHar), 128.7 (CHar), 127.0 (CHar), 124.0 (CHar), 114.2 (CHar), 66.4 (CH2), 55.3 (OCH_3), 14.2 (CH_3)**.**

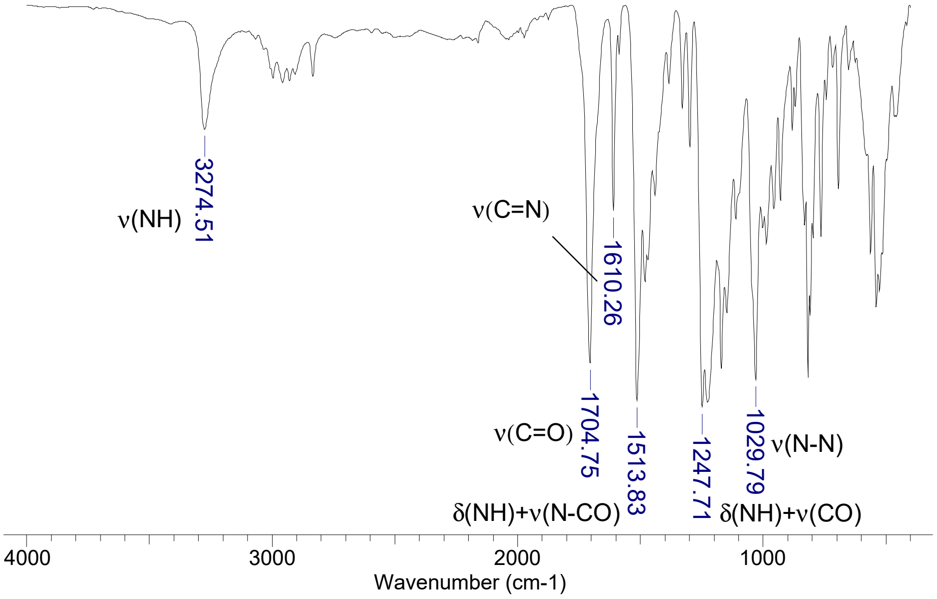

Figure S4. Infrared spectra the Schiff base ligand H_2L**.**

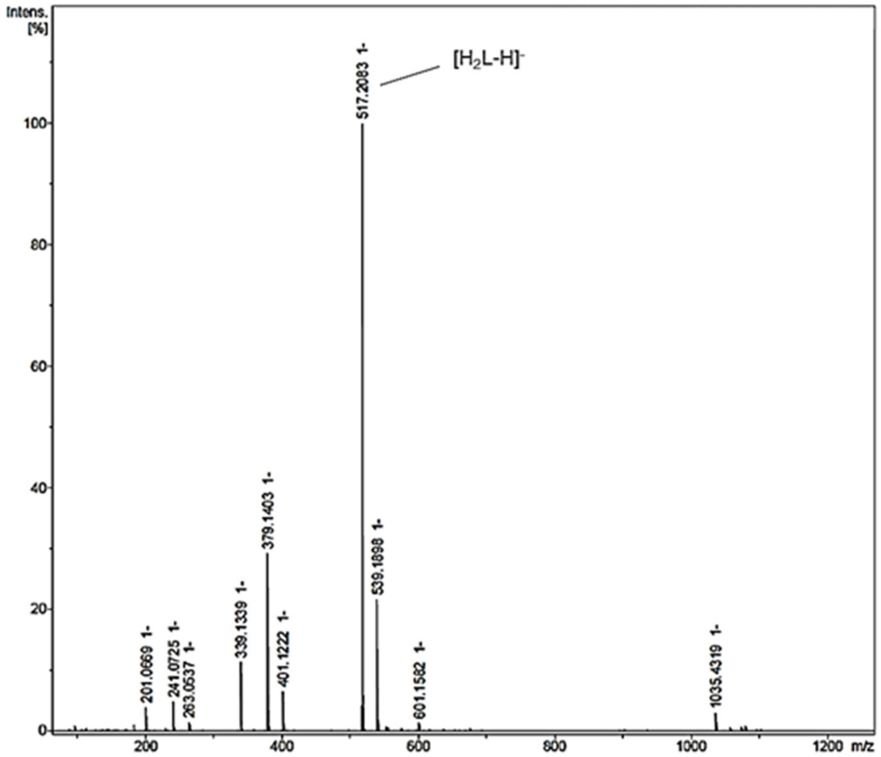

Figure S5. Mass spectra of the Schiff base ligand H_2L

2. Copper(II) complex [$Cu_2(L^1)(OH)$]

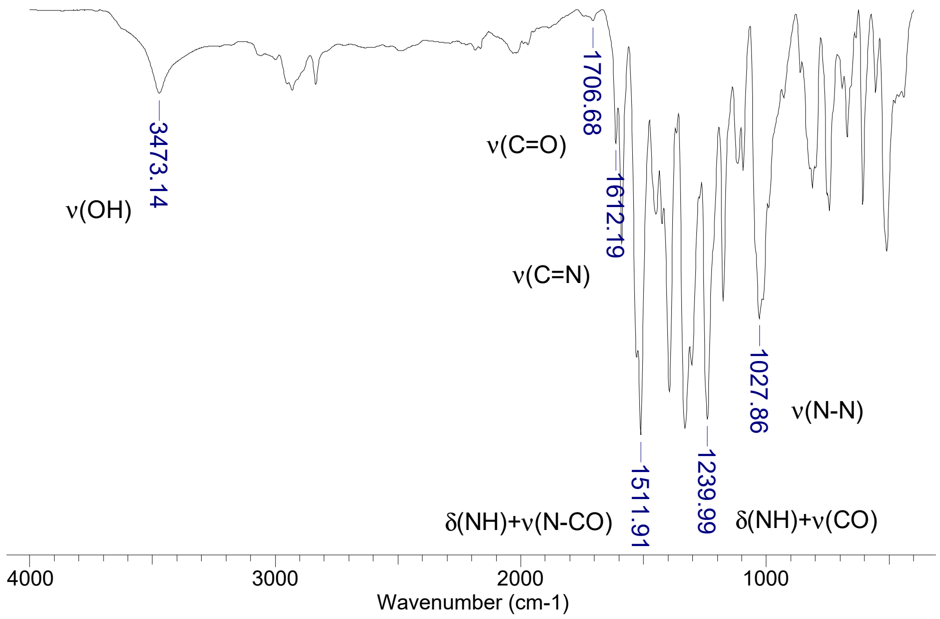

Figure S6. Infrared spectra of [$Cu_2(L^1)(OH)$].

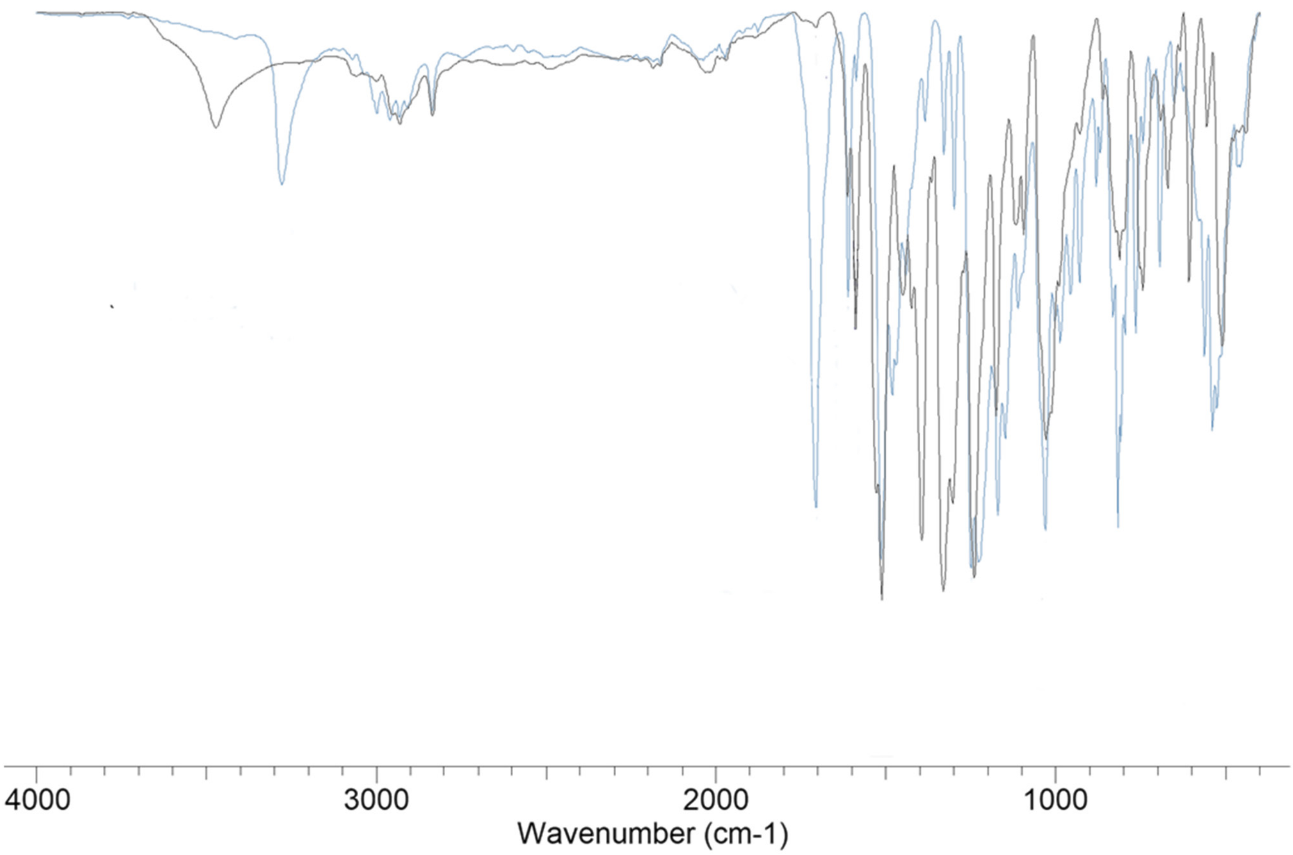

Figure S7. Superposition of the infrared spectra of the copper complex [$Cu_2(L^1)(OH)$] (black) and the H_2L ligand (blue).

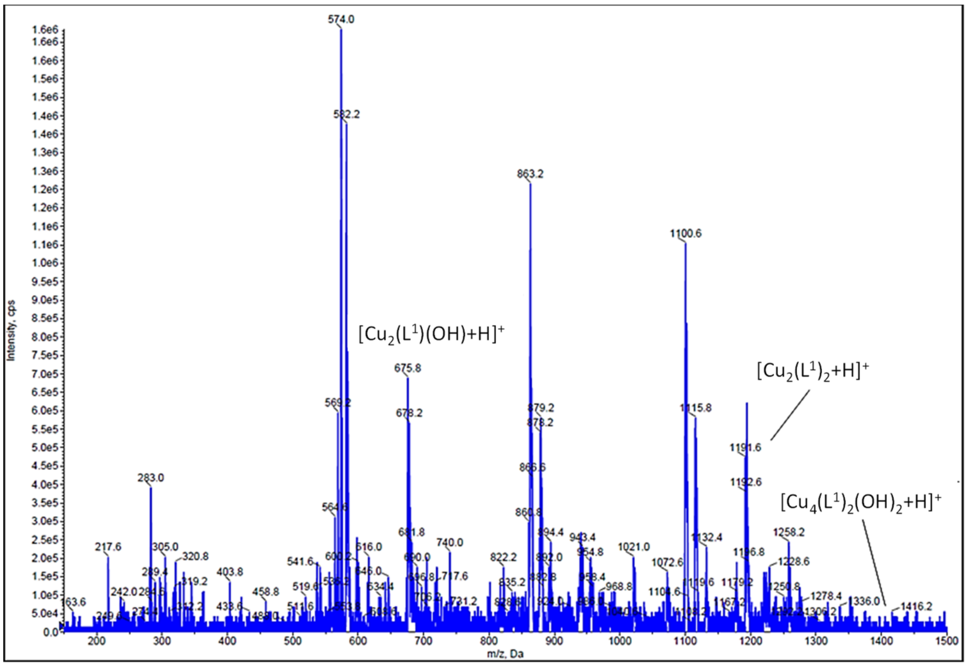

Figure S8. Mass spectra of the $[Cu_2(L^1)(OH)]$.

Table S1. Main crystallographic data for $[Cu_2(L^1)(OH)]\cdot 2CH_3CN$.

	$[Cu_2(L^1)(OH)]\cdot 2CH_3CN$
Molecular formula	$Cu_4C_{60}H_{62}N_{10}O_{16}$
Molecular weight	1433.36
Spatial group	*P-1*
Crystalline system	Triclinic
Crystal size/mm	0.15 × 0.07 × 0.03
a/Å	9.0922(2)
b/Å	11.8957(3)
c/Å	14.0610(4)
α/º	94.0500(10)
β/º	90.3190(10)
γ/º	103.8490(10)
Temperature/K	100
Volume/Å^3	1472.52(7)
Z	1
Measured reflexions	32274
Unique reflexions [R_{int}]	8768 [0.0421]
μ/mm^{-1}	1.505
Residues/e Å^{-3}	-0.49 and -0.53
R	0.0829
*w*R	0.0771

Table S2. Main bond distances and angles for $[Cu_2(L^1)(OH)]\cdot 2CH_3CN$.

Bond distances (Å)					
Cu1-O39	1.9104(17)	Cu1-O28	1.9185(15)	C27-O28	1.2770(3)
Cu1-O40	1.9234(15)	Cu2-N9	1.9145(19)	C27-N26	1.3160(3)
Cu2-O39	1.9371(17)	Cu2-O12	1.9524(15)	O39-Cu2i	2.3311(17)
Cu2-O40	1.9498(15)	N9-N10	1.3990(2)	Cu2-O39i	2.3311(17)
Cu1-Cu2	2.9233(4)	N25-N26	1.4020(2)	Cu1-O12i	2.6780
C1-O40	1.3340(3)	C11-O12	1.2880(3)	O12-Cu1i	2.6780
Cu1-N25	1.9105(19)	C11-N10	1.3050(3)		
**Bond angles (^{0})**					
O39-Cu1-N25	174.57(8)	O40-Cu1-N25	92.66(7)	O28-Cu1-Cu2	141.88(5)
O39-Cu1-O28	101.66(7)	O40-Cu2-O12	173.46(7)	O40-Cu1-Cu2	41.34(4)
O39-Cu1-O40	82.00(7)	O40-Cu2-N9	91.13(7)	O39-Cu2i-O40i	99.06(6)
O39-Cu2-O40	80.64(7)	N25-Cu1-O28	83.73(7)	O39-Cu2i-N9i	107.84(7)
O39-Cu2-N9	169.70(8)	N9-Cu2-O12	83.70(7)	O12-Cu2-O39i	86.37(6)
O39-Cu2-O12	104.03(7)	O39-Cu1-Cu2	40.90(5)	O39-Cu2-O39i	79.78(7)
O40-Cu1-O28	175.02(7)	N25-Cu1-Cu2	133.85(6)		
